# Hip fracture or not? The reversed prevalence effect among non-experts’ diagnosis

**DOI:** 10.1186/s41235-022-00455-w

**Published:** 2023-01-05

**Authors:** Hanshu Zhang, Shen-Wu Hung, Yu-Pin Chen, Jan-Wen Ku, Philip Tseng, Yueh-Hsun Lu, Cheng-Ta Yang

**Affiliations:** 1 School of Psychology, Central China Normal University, Wuhan, Hubei China; 2 Department of Orthopedics, Wan Fang Hospital, Taipei Medical University, Taipei, Taiwan; 3 Department of Orthopedics, School of Medicine, College of Medicine, Taipei Medical University, Taipei, Taiwan; 4 Department of Radiology, Shuang-Ho Hospital, Taipei Medical University, No. 291, Zhongzheng Rd., Zhonghe Dist., New Taipei City, Taiwan; 5 Graduate Institute of Mind, Brain, and Consciousness, Taipei Medical University, Taipei, Taiwan; 6 Department of Radiology, School of Medicine, College of Medicine, Taipei Medical University, Taipei, Taiwan; 7 Department of Psychology, National Chung-Kung University, Tainan, Taiwan

**Keywords:** Prevalence effect, Medical image perception, Hip fracture diagnosis

## Abstract

Despite numerous investigations of the prevalence effect on medical image perception, little research has been done to examine the effect of expertise, and its possible interaction with prevalence. In this study, medical practitioners were instructed to detect the presence of hip fracture in 50 X-ray images with either high prevalence (*N*_signal_ = 40) or low prevalence (*N*_signal_ = 10). Results showed that compared to novices (e.g., pediatricians, dentists, neurologists), the manipulation of prevalence shifted participant’s criteria in a different direction for experts who perform hip fracture diagnosis on a daily basis. That is, when prevalence rate is low (*p*_fracture-present_ = 0.2), experts held more conservative criteria in answering “fracture-present,” whereas novices were more likely to believe there was fracture. Importantly, participants’ detection discriminability did not vary by the prevalence condition. In addition, all participants were more conservative with “fracture-present” responses when task difficulty increased. We suspect the apparent opposite criteria shift between experts and novices may have come from medical training that made novices to believe that a miss would result in larger cost compared to false positive, or because they failed to update their prior belief about the signal prevalence in the task, both would suggest that novices and experts may have different beliefs in placing the optimal strategy in the hip fracture diagnosis. Our work can contribute to medical education training as well as other applied clinical diagnosis that aims to mitigate the prevalence effect.

**Supplementary Information:**

The online version contains supplementary material available at 10.1186/s41235-022-00455-w.

## Introduction

Expertise influences medical image perception (e.g., Manning et al., 2006; Waite et al., 2019; Wood et al., 2013). Previous studies have shown that experts employ a holistic and global processing strategy in screening medical images (Kundel et al., 2007), whereas novices are more distracted by irrelevant regions (Brunye et al., 2014). Despite this discrepancy in detection ability, research has found that both experts and novices can suffer from the prevalence effect (e.g., Nakashima et al., 2015)—a phenomenon in visual search where more misses are observed when targets are rare (Wolfe et al., 2005). In this study, we further investigate whether these miss errors are influenced by task difficulty as a function of observers’ expertise. Medical practitioners, both experts (e.g., radiologists, orthopedicians) and novices (e.g., pediatricians, dentists, neurologists), in hip fracture were shown X-ray images and asked to indicate whether hip fracture is present or not in every image. Our data indicate an opposite effect in criterion shift between experts and novices: Where experts became more conservative when there were fewer targets, novices were more likely report “fracture-present” in the same target-rare condition (i.e., the low prevalence). Therefore, factors such as learning, memory, and expectations that shape experts’ top-down medical image perception (Wolfe et al., 2016) also influence the prevalence reports.

Even highly trained experts are susceptible to the prevalence effect. However, as reviewed by Horowitz (2017), the existing research provides few observations for discussing the interaction between the prevalence effect and expertise. Gur et al. (2003) tested three groups of residents, fellows, and board-certified radiologists in reporting abnormalities in chest images. Their results did not show interactions between expertise and performance and thus concluded that the prevalence effect only has a marginal existence. Nakashima et al. (2013) tested radiologists and novices in the context of multi-target search scenarios. The overall target prevalence was 50% in which the prevalence of each target was further decided by the severity of illness (i.e., bulla: 40%, ground-glass nodule: 8%, cancer: 2%). Their results indicated that compared to (true) novices’ performance, radiologists produced a higher miss rate for non-serious lesions than for serious lesions. With only target severity (cancer and bulla) and prevalence rate (50% and 2%) considered, Nakashima et al. (2015) found that cancer detection by radiologists was higher than that in bulla detection but no difference in novices. Together, these two studies suggest that experience and depth of knowledge are important aspects of radiologists’ skills.

In addition to the lack of evidence in testing the interaction between the expertise and prevalence effect, there is also very few studies investigating how the prevalence effect can impact applied medical diagnosis. Evans et al., 2013 introduced a novel search scenario where radiologists were asked to search in their daily work compared to search in a laboratory setting. Their results indicated that these medical experts missed much more cancers in daily practices compared to the high-prevalence laboratory setting. Therefore, prevalence effect may be a real factor in clinical misdiagnoses. Yet, most studies on this topic to date have either recruited radiologists in small sample sizes (e.g., Gur et al., 2003; Nakashima et al., 2013, 2015) or instructed their non-experts to search for simple shapes to infer experts’ search performance in the medical search scenario (e.g., Lau & Huang, 2010; Peltier & Becker, 2016; Schwark et al., 2012, 2013).

In this study, we introduced radiographs of hip fracture as a model to investigate the possible interaction between prevalence and expertise to mitigate the issues discussed above. As the population ages, the incidence of hip fracture is increasing and is expected to reach 4.5 million worldwide by year 2050 (Cooper et al., 2011), evidenced by an 8.6% increase based on the insurance report in Taiwan (Chen et al., 2015). The unique scenario with the hip fracture diagnosis is that most hip fracture patients are first diagnosed at the emergency department (ED) because they experience pain and difficulty in walking after acute trauma, whereas very few patients are initially presented to the outpatient department (OPD) instead of ED. This difference in diagnosis probability between ED (high prevalence) and OPD (low prevalence) thus may contribute to a prevalence effect in the real-world medical setting. Importantly, residents in ED have been shown to be more prone to misdiagnosis than experienced physicians (Leeper et al., 2013). Considering that up to one-third of the older adult patients with hip fractures would exhibit permanent functional loss and severe dependence at 1-year follow-up (Chen et al., 2021), it is essential to explore the influence of prevalence effect on hip fracture diagnosis as a function of medical practitioners’ experience to prevent the fracture from being initially missed. Furthermore, in this study we recruit non-experts who are also medical practitioners (e.g., pediatricians, neurologists), but not in the domain of hip fracture, to make fairer comparisons between experts and non-experts (i.e., novice).

To conclude, the present study aims at exploring medical practitioners’ hip fracture diagnoses in the context of high-prevalence versus low-prevalence conditions. First, we expect an interaction between expertise and prevalence effect. Given that most previous research emphasizes the criteria shift of the expert group (e.g., Evans et al., 2013), we expect that this mechanism should also apply to our experts’ performance, whereas novices who do not have daily experience in diagnosing hip fractures may reply more on their prior belief about the experimental setting. In addition, we also justify the discriminability between the experts and novices by further examining the influence of task difficulty on the prevalence effect.

## Methods

### Participants

Participants were recruited through email invitations and online advertisements (e.g., online forums, online chat groups, etc.). In total, 106 participants (*N*_female_ = 62) completed the high-prevalence condition, and 85 participants (*N*_female_ = 53) completed the low-prevalence condition, with some participants (*N* = 58) completing both conditions. Participants’ professional experience ranged from medical students to attending physicians of varying years of experience. Given that previous research did not provide a clear classification in defining experts and novices in the medical image reading domain (Nakashima et al., 2013; Nocum et al., 2013), in the current study, radiologists, orthopedists, and emergency physicians who had access to hip fracture in their daily diagnosis were considered experts, and all others (i.e., physicians of other specialties or medical students) were considered novices. This leads to a total of 38 experts’ observations in the high-prevalence condition and 31 experts’ observations in the low-prevalence condition.

### Design and material

Eighty-five hip fracture radiographs were collected from a prospective hip fracture registry at a single medical center in Taipei, Taiwan. This hip fracture registry, which was approved by the Ethics Committee of Taipei Medical University TMU-JIRB N201709053, prospectively collected clinical data and hip radiographs from 777 patients (age ≥ 60 years) who underwent surgery for hip fracture since January 1, 2017. All images were anonymized, and any text information on the image (e.g., date) was cropped. From this registry dataset, 45 radiographs of femoral neck fractures were selected as test images. Another group of 40 normal hip radiographs from elder adults (age ≥ 60 years) without hip fractures were selected from the same medical center as the healthy control images. This total of 85 radiographs were allocated into 2 sets, where one set contained 40 fracture images and 10 healthy images (the high-prevalence set), and the other contained 10 fracture images and 40 healthy images (the low-prevalence set). Ten healthy images and 5 fracture images (1 easy, and 4 medium, elaborated below) were shared between both versions.

**Fig. 1:**
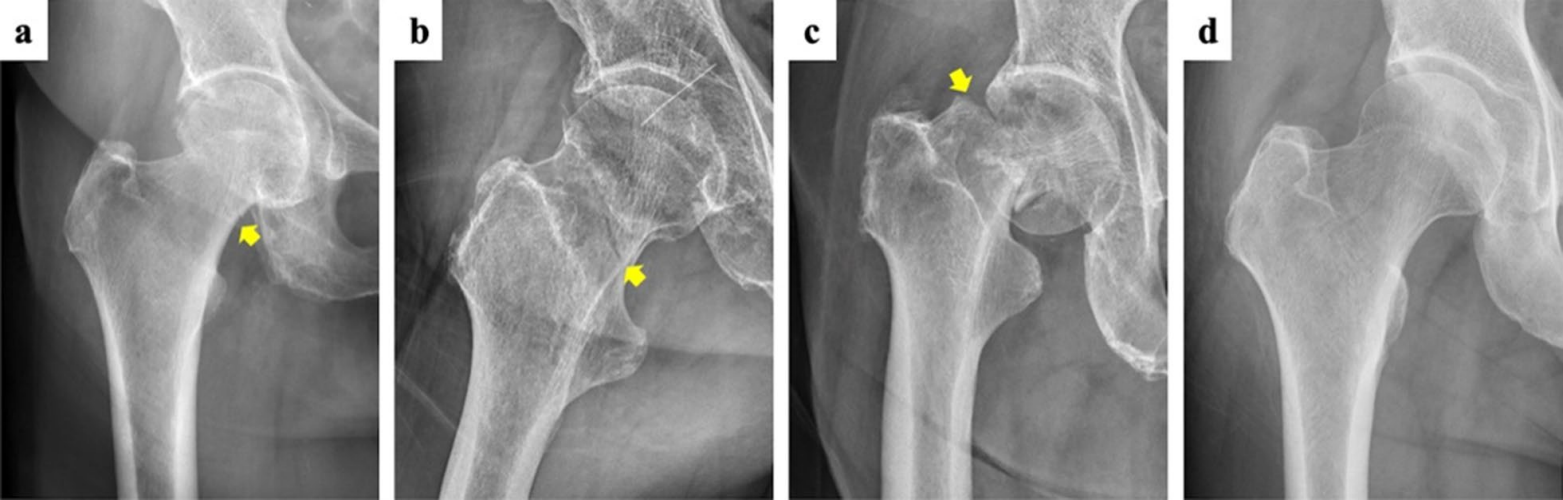
Sample X-ray images. Images of varying levels of difficulty, as judged by our expert radiologist and orthopediatrician. There were three levels of difficulty: difficult (level III, **a**), medium (level II, **b**), easy (level I, **c**), and healthy images as the control (**d**). Arrow indicates the fracture line (not shown to the participants)

The 45 hip fracture radiographs were categorized into 3 levels of difficulty by three well-trained experts by two authors, including an orthopedic surgeon (Y.-P. Chen) and a radiologist (Y.-H. Lu), who both have more than 20 years of experience in reading and interpreting hip radiographs. The two experts were independently consulted to define the hip fracture radiographs. Disagreements were resolved by consensus, and if consensus was not achieved, a third expert majoring in the subspecialty of ortho-radiology (J.-W. Ku) was incorporated to make an arbitration decision. Among the 45 fracture images, this resulted in 14 easy, 15 medium, and 11 difficult fracture radiographs (see Fig. 1 for an example). Notably, 8 of the 11 difficult radiographs came from hip fracture patients who were initially missed at their first presentation to the emergency department.

### Procedure

Links to both the high- and low-prevalence sets were sent to the participants online. Participants were asked to complete either set or both, in their own time. Participants were not informed of the purpose of the experiment and were not instructed to complete the sets in any particular order. After completing the task, the correct diagnosis answers were sent to participants upon request.

Both high- and low-prevalence sets contained 50 images, with an 80% positive rate in the high-prevalence set and 20% in the low-prevalence set. Within each set, the order of the 50 images was initially randomized, and the same randomized order was used for all the participants. All images were displayed in one column/page for the participants to scroll through. With each image, participants were instructed to select 1 out of the 5 available responses: “No fracture—very sure,” “No fracture—unsure,” “Not sure,” “Fracture—unsure,” and “Fracture—very sure.” These radial buttons were placed from left to right in the same order, with “Do not know” in the middle. There was no time limit for the individual questions or the set as a whole. Toward the end of each set, participants were asked for their field of specialty (e.g., radiology, orthopedics, etc.), years of professional experience (e.g., 2nd year resident, 5th year visiting physician, etc.), sex, and the kind of device they used for completing the task (e.g., phone, tablet, computer).

## Results

**Fig. 2:**
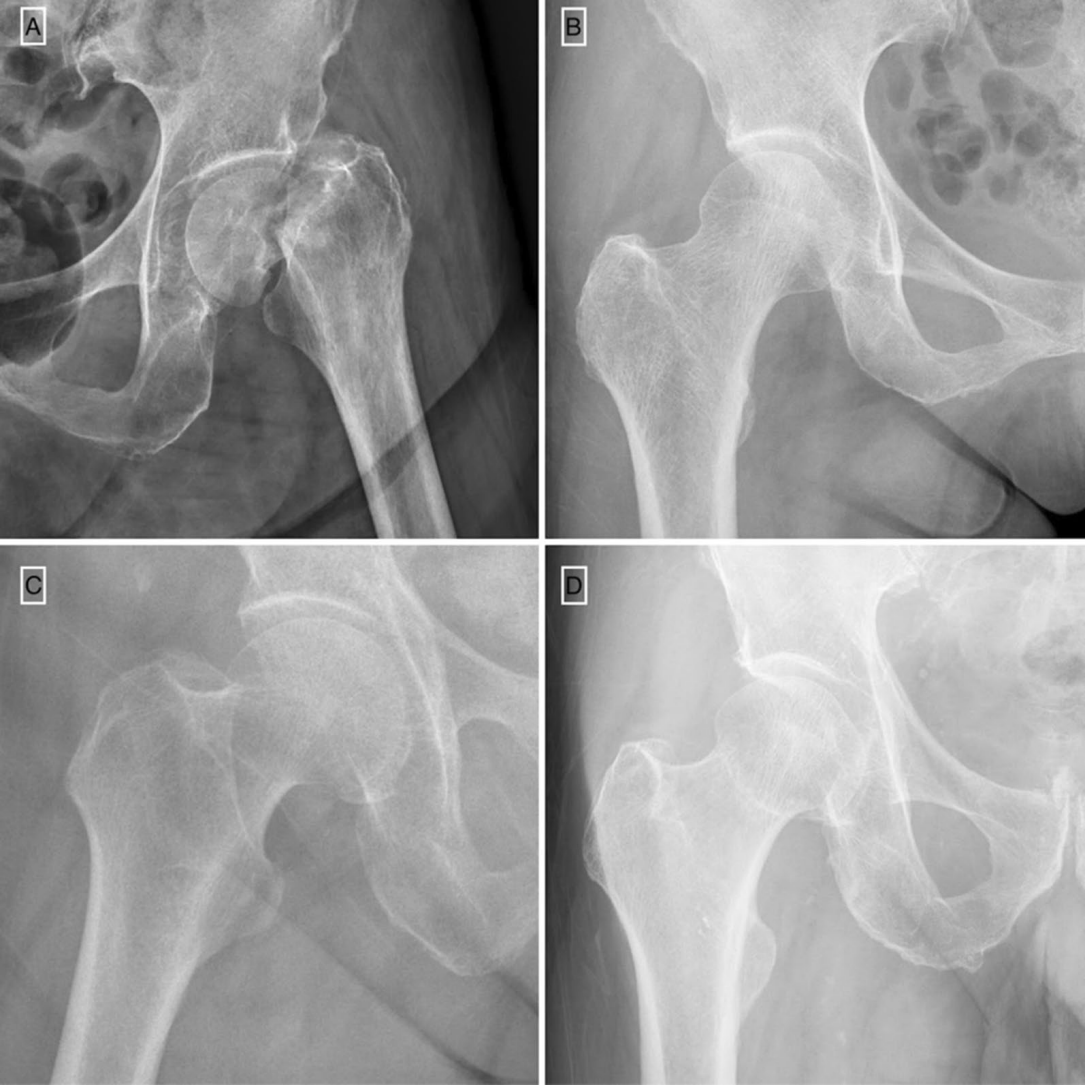
Sample X-ray images of different diagnosis rates. **A**: The significant hip fracture with a high detection rate of 98.3%; **B** The normal and healthy hip with 98.9% correct rejection; **C** The non-fracture hip but the sclerotic line causes a high false positive rate, 67.4%; and **D** An occult hip fracture without displacement. The correction detection of fracture is only 13.6%

Figure 2 presents examples of different diagnosis accuracy.^1^ Fracture in Fig. 2A causes significant displacement. Therefore, it has the highest true positive rate of 98.3%. Figure 2B shows a normal and healthy hip in the young-aged case. The homogeneous bony condition causes the highest true negative rate of 98.9% for both experts and novices. The sclerotic line of the right femoral neck in Fig. 2C confuses the interpreter and causes a high false positive rate of 67.4%. Figure 2D demonstrates occult non-displaced fracture, which is the hardest scenario to detect, resulting in a low detection rate of 13.6% in our study. Because we tried to include as many medical practitioners as possible in our initial recruiting stage, we did not restrict the physicians’ specialists. Therefore, we excluded participants whose accuracy was below 60% to balance the level of expertise knowledge (for participants who were dentist, pediatricians, family care doctors, etc.) for recruited participants in our study. This leaves us with 96 observations (*N*_experts_ = 38) in the high-prevalence condition and 70 observations (*N*_experts_ = 28) in the low-prevalence condition.

Participants’ correct and error responses were recorded and analyzed. We assessed the effects of difficulty and prevalence levels on performance, in terms of the decision criteria and detection discriminability (*d’*) (Wolfe & Van Wert, 2010, Dual Threshold Model) by applying Fuzzy Signal Detection Theory (SDT; Parasuraman et al., 2000). Because there was a limited number of signal-present trials (*N* = 10) in the low-prevalence condition, we did not further separate it into different difficulty levels. As a result, we only tested the influence of difficulty for the high-prevalence level. We conducted a one-way ANOVA^2^ analysis using JASP (JASP Team, 2022), and Tukey was used for post hoc pairwise comparisons.

The Fuzzy SDT follows the same logic as the regular one. The rates for each of the four decision responses (HR: hit rate, MR: miss rate, FAR: false alarm rate, and CRR: correct rejection rate) can be calculated by summing each response category and dividing it by the total number of each associated signal or non-signal trials. The detection discriminability is estimated by *d*’ = *z*(HR) − *z*(FAR): It defines how well participants are able to discriminate between signals from non-signal trials. A higher *d*’ means better detection discriminability, whereas a negative d’ indicates that participants entirely mix up signals with non-signals. The criteria *c* =  − [*z*(HR) − *z*(FAR)]/2, representing the relative bias in responding signal-present (“yes”) and signal-absent (“no”). When *c* = 0, the bias is equal; the higher *c*, the more conservative criteria that participants in answering signal-present—resulting in more observations in correct rejections but also more misses. Specifically, we also apply the correction proposed by Berkson (1953) for extremes of 0 or 1 correction to avoid infinite *z*(HR) or *z*(FAR). When the response rate *p* is 0, *p* is corrected as *p* = 1/(2 $\times \hspace{0.17em}$*N*) and *p* = 1 − (1/2 $\times \hspace{0.17em}$*N*), with *N* equal to the number of signals (for *p*_HR_) or non-signals (for *p*_FAR_) observations.

The exception in the Fuzzy SDT is that it can treat stimuli and (or) response as continuous variables. To map the discrete ratings of confidence levels, participants’ responses are equally separated into 1 (fracture—very sure), 0.75 (fracture—unsure), 0.5 (not sure), 0.25 (no facture—unsure), 0 (no facture—very sure) 5 levels and the existence of signal (s) corresponds to 1. In this way, for a signal-present (hip fracture; *s* = 1) trial, if the participant’s response *r* is 0.75 (fracture—unsure), the trial will count as 0.75 hit, while it also indicates 0.25 miss. In the case that the participant’s responses are 0.5 (not sure), it will be treated as 0.5 miss and 0.5 hit in a signal-present trial and it will be treated as 0.5 false alarm and 0.5 correct rejection in a signal-absent trial.

The four response categories, hit, miss, false alarm, and correct rejection, are therefore defined by the following equation. The overall result is the list in Table 1.

$${\text{Hit}}:{\text{ H }} = {\text{ min }}\left( {s, \, r} \right)$$

$${\text{Miss}}:{\text{M}} = {\text{max }}\left( {s - r, \, 0} \right)$$

$${\text{False alarm}}:{\text{FA}} = {\text{max }}\left( {r - s,\;0} \right)$$

$${\text{Correct rejection}}:{\text{CR}} = {\text{min }}\left( {1{-}s, \, 1{-}r} \right)$$

**Table 1:** The summary of SDT results in the experiment for experts and novices

	High prevalence		Low prevalence	
	Experts	Novices	Experts	Novices
*d*’	1.68 (0.54)	1.29 (0.46)	1.63 (0.46)	1.32 (0.49)
Criteria	− 0.07 (0.35)	0.004 (0.36)	0.053 (0.40)	− 0.11 (0.34)
Miss rate (%)	19.67 (9.29)	27.90 (11.38)	23.58 (11.50)	24.64 (13.29)
FA rate (%)	24.73 (12.54)	27.67 (12.09)	22.17 (10.47)	29.91 (9.24)

SDs in parenthesis

### The effect of prevalence

**Fig. 3:**
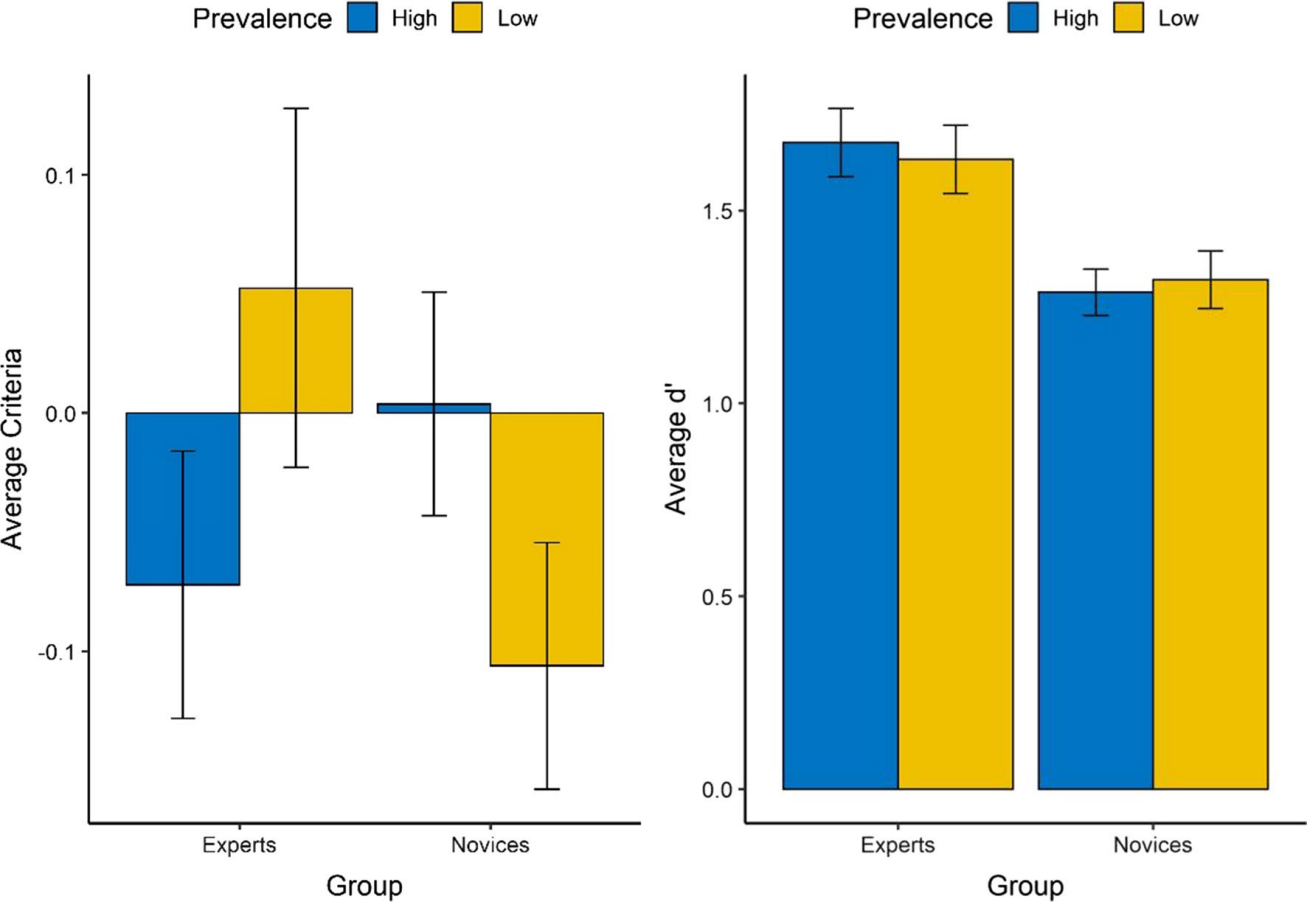
The average criteria and d’ for the group of experts and novices as a function of the level of prevalence. The error bar represents ± one standard error

We tested whether novices and experts’ performance differed in low and high prevalence. Presumably, the conventional prevalence effect assumed that participants were biased to answer “signal-absent” in the low-prevalence condition; that is, they were more likely to miss the signal when the signal was rare. Figure 3 describes the average criteria and detection discriminability (*d*’) based on the group and prevalence level. As hypothesized, experts had more liberal criteria when the prevalence was high; thus, they were more likely to answer “signal-present.” The interesting finding is that such an ordered relationship is reversed for the novice group: Novices had more liberal criteria when the prevalence was low, resulting in more “signal-present” responses. In addition, the detection discriminability did not vary by the prevalence level, and experts consistently had better performance in identifying signals (represented by a higher* d*’) compared to novices.

One-way ANOVA on criteria indicated that there was only a significant interaction effect between the group and prevalence, *F* (1, 162) = 4.19, *p* = 0.04, ${\eta }_{p}^{2}$ = 0.03, whereas the main effects did not reach the significance level for the group (*F* (1, 162) = 0.52, *p* = 0.47) or the prevalence level (*F* (1, 162) = 0.02, *p* = 0.90). We notice that when the prevalence level changed from high to low, experts’ criteria became more conservative (thus more observed “signal-absent” responses), whereas novices’ criteria became more liberal, despite that post hoc pairwise interactions did not reach the significance level with the Tukey correction.

### The effect of difficulty levels

**Fig. 4:**
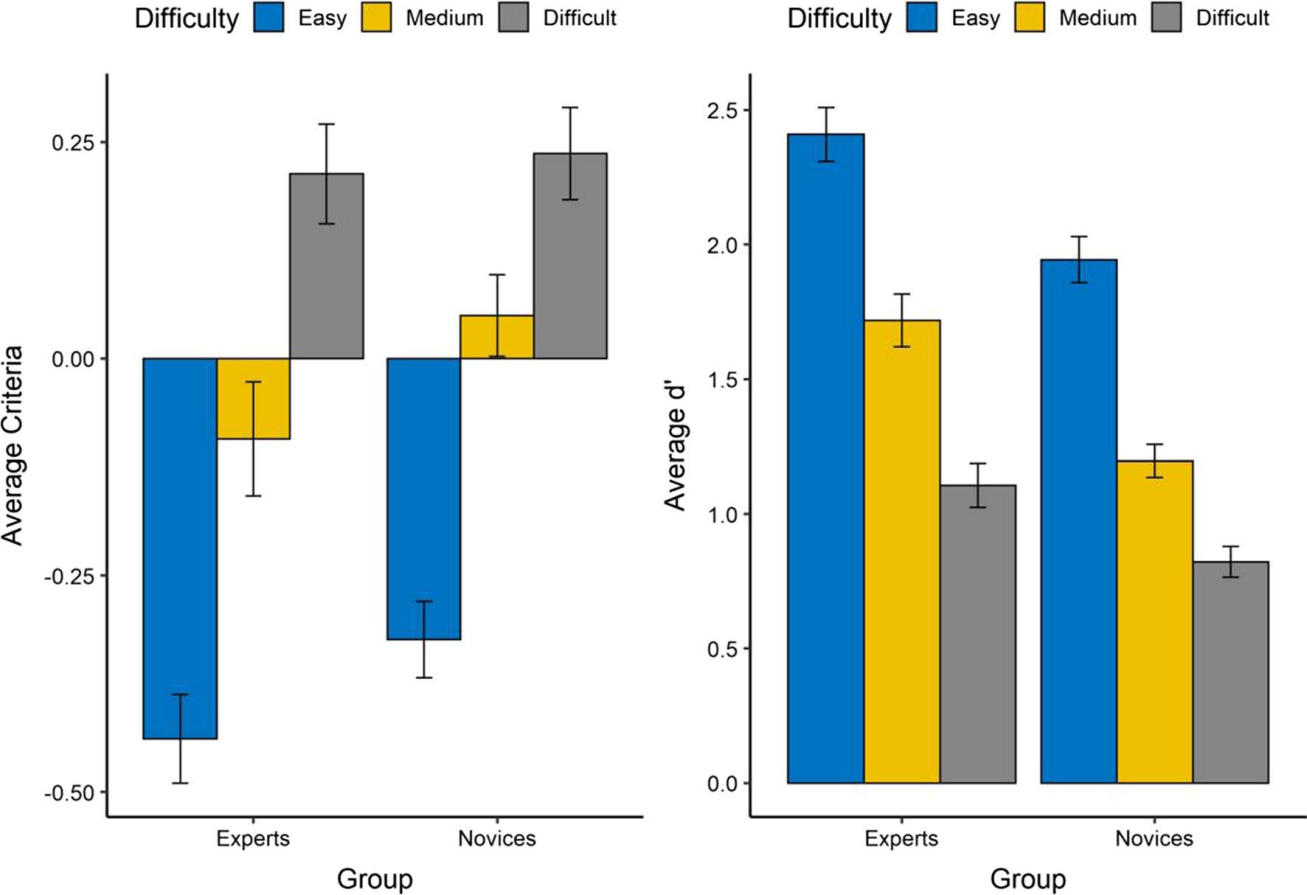
The average criteria and *d*’ for the group of experts and novices as a function of the levels of difficulty in the high-prevalence condition. The error bar represents ± one standard error

Figure 4 represents participants’ criteria and detection discriminability as a function of difficulty levels in the high-prevalence condition. As the figure shows, both experts and novices had better detection discriminability (*d*’) at the easy level compared to the medium and difficult levels. Therefore, in the medium-level condition, novices were more likely to make a conservative decision, whereas experts tended to make a liberal decision.

One-way ANOVA on participants’ criteria results indicated that there was a main effect of difficulty levels (*F* (2, 282) = 17.08, *p* < 0.001, ${\eta }_{p}^{2}$ = 0.34), where participants adopted more conservative criteria in the difficult condition than they did in the medium (*t* = 4.58, *d* = 0.68, *p* < 0.001) and easy condition (*t* = 11.26, *d* = 1.66, *p* < 0.001). In the same vein, participants’ criterion in the medium condition was also more conservative than those in the easy condition (*t* = 6.69, *d* = 0.99, *p* < 0.001). There was also a main effect of the group (*F* (1,282) = 4.51, *p* = 0.034, ${\eta }_{p}^{2}$ = 0.016). Overall, novices had slightly more liberal criteria compared to experts (*t* = 2.13, *d* = 0.26, *p* = 0.034). There was no statistically significant interaction between the group and difficulty (*F* (2, 282) = 0.664, *p* = 0.52).

Similarly, one-way ANOVA on participants’ d’ indicated that there was a main effect of the difficulty level (*F* (2, 282) = 114.55, *p* < 0.001, ${\eta }_{p}^{2}$ = 0.45). Participants in the difficulty condition had worse perceptual discriminability than the medium (*t* = 6.12, *d* = 0.90, *p* < 0.001) and easy condition (*t* = 15.05, *d* = 2.21, *p* < 0.001), with the medium condition also resulting in worse detection discriminability compared to the easy condition (*t* = 8.93, *d* = 1.32, *p* < 0.001). There was also a main effect of the group (*F* (1, 282) = 41.42, *p* < 0.001, ${\eta }_{p}^{2}$ = 0.13), and experts had better detection discriminability compared to novices (*t* = 6.44, *d* = 0.78, *p* < 0.001). There was no significant interaction between the main factors (*F* (2, 282) = 1.19, *p* = 0.31).

## Discussion

In this study, medical practitioners viewed different hip radiographs in sequence and judged whether a hip fracture was present or not for each image. We observed an interaction between expertise and effect, where opposite trends of criteria shifts were found for experts and novices. Consistent with Wolfe and Van Wert (2010), experts’ criteria were more conservative (thus biased toward “fracture-absent”) in the low-prevalence condition (pfracture-present = 0.2) compared to the high-prevalence condition (pfracture-present = 0.8). Nevertheless, this type of criteria shift only applies to experts’ diagnoses. For novices who did not have experience in hip fracture diagnosis every day, they were more likely to respond “fracture-present” when the prevalence was low. In addition, our study reveals that the discriminability d’ does not change as a function of the prevalence, which is also consistent with previous study by Wolfe et al. (2007).

What makes an expert? To explore this question, we also focused on the effect of task difficulty on participants’ performance in the high-prevalence condition. When the image judgment was difficult, both experts and novices tended to respond “signal-absent” and thus missing the hip fracture. While the difficulty-based criteria shift can be a statistical artifact, another possibility may be that participants are using different cues to make responses, which coincidentally corresponds to our levels of task difficulty. In the easy condition, there are global spatial cues for participants to utilize (e.g., the obvious dislocation, Fig. 1d), whereas in the difficult condition, participants may need to rely more on specific local information. For example, the fracture line in Fig. 1a overlaps with the normal structure in the radiograph, resulting in an uncommon scenario in clinical practice for participants to make the judgment. Interestingly, although we observed a significant main effect of task difficulty, there was no significant interaction between expertise and task difficulty (Fig. 4). We suspect this may be specific to our study since our novices are also medical professionals who have experience with radiographs but just not of hip fracture on the daily basis. In addition, given that task difficulty does not exactly correspond with illness severity, future research should further explore tasks that can differentiate experts and novices with illness severity accounted for in a balanced search scenario (c.f., Nakashima et al., 2013).

Although most studies to date have focused on chest X-rays, with an emergence of more medical areas in recent years, the present study demonstrates that the prevalence effect can also be applied to expert diagnosis of hip fracture. Therefore, one question that arises is why the prevalence effect seems to be very persistent and highly generalizable in medical image perception. We think the experts’ ability to detect the prevalence and adjust response criteria accordingly may have been deeply reinforced through thousands of hours of implicit learning (e.g., Tseng et al., 2010, 2011). Indeed, in medical image processing, implicit detection of breast cancer by expert radiologists has also been documented (Brennan et al., 2018), presumably due to some rapidly extracted gist information via the nonselective pathway (e.g., Sampanes et al., 2008), which allows expert radiologists to make above-chance guesses even with less than one second of image exposure (i.e., no explicit detection). Importantly, because of these experts’ daily exposure to real-life prevalence rates, experts may react differently to our prevalence manipulation due to their experience with the real-life base rate of hip fractures. In contrast, novices who do not have experience with real-life base rates might easily converge to a 50/50 base rate due to their lack of accumulated experience and are therefore much more easily led (or misled) by our prevalence manipulation. In this light, “expertise” is simply defined by the amount of domain-specific exposure that is accumulated throughout years of experience. Although our current study fails to establish a correlation between participants’ years of professional experience and criteria (shift), preliminary analysis with participants’ years of professional experience does show that our observed reversal in criteria shift from the novice group is more evident among the juniors (≤ 10 years of experience) and not seniors (> 10 years). Therefore, it is possible that our results from the experts were mainly driven by an implicitly accumulated baseline probability of hip fracture, or illness in general that these practitioners have encountered in daily life. We plan to explore the criteria shift with different daily diagnosis base rates for practitioners’ (e.g., dermatologists, respiratory physicians, pediatricians, etc.) in our future studies.

One important control in our study is the use of medical practitioners (e.g., pediatricians, dentists, neurologists) who were trained but have no access to daily fracture diagnosis as our novices, as opposed to using genuine novices or medical students (e.g., Gur et al., 2003; Nakashima et al., 2015). These non-experts may not only differ in their discriminability for the fracture but also in how they interpret the probability of “hip fracture” in our current diagnosis scenario. As discussed above, novices may have different prior beliefs compared to experts in adjusting their expectations during the task. In this specific study with medical practitioners, we also suspect this phenomenon can be induced by the believed cost for false alarm and miss for these participants: False alarm can be far less costly compared to a miss (legally speaking in the health industry). Therefore, medical practitioners may be more liberal about their response when the prevalence is low (c.f., Navalpakkam et al., 2009) given their limited exposure to the fracture diagnosis. In addition, viewing conditions may also lead to different performances since experts can have access to better-quality monitors for diagnosing. Future studies can further test whether this reversal in criteria shift only applies to this unique setting or can also be found in other clinical diagnosis scenarios. Previous research has explored various possibilities to accelerate training from novice to expert. For example, Litchfield et al. (2010) identified that showing novice radiologists with the search behavior of either a naïve or expert radiologist can temporarily improve their diagnostic performance. Therefore, we expect our work can also contribute to this training process by utilizing the criteria shift discrepancy, such as offering a different information access (Zhang & Houpt, 2020).

## Conclusion

The current study examines the influence of expertise and difficulty on medical practitioners’ hip fracture diagnostic performance. Our results indicate that the bias in answering “fracture-present” depends very much on the prevalence context that medical practitioners are presented with. Specifically, experts held more conservative criteria in the low-prevalence condition, whereas novices showed the opposite pattern and were more likely to believe there is a fracture in the same low-prevalence condition. In addition, our task also reveals an influence of task difficulty, where all medical practitioners are more conservative in indicating a hip fracture when the judgment becomes difficult. We believe this line of research can contribute to medical education and training, as well as other applied clinical diagnoses to mitigate the prevalence effect.

## Supplementary Information

**Additional file 1.** A group of participants have participated in both studies, and were treated as unique observations in the ANOVA analysis for the prevalence effect. Alternative ANOVA analyses are provided here with different combinations of unique observations.

## Data Availability

This study was not preregistered. All experimental stimuli, data, and analysis code have been made publicly available at Open Science Framework (OSF) and can be accessed at https://osf.io/meagk/.

## Footnotes

1 To get a basic understanding of how well participants perform the task, we consider the responses of “Fracture—unsure” and “Fracture—very sure” given the signal trials (i.e., when hip fracture exists) and responses of “No fracture—very sure” and “No fracture—unsure” given the non-signal trials as correct. Note this is different from how we apply the signal detection theory in data analysis.

2 Despite a group of participants having participated in both studies, they were treated as unique observations in the ANOVA analysis for the prevalence effect. Additional file 1 includes alternative ANOVA analyses with different combinations of unique observations.
