# Supplementary material for: Hip fracture or not? The reversed prevalence effect among non-experts’ diagnosis
Source: Cogn Res Princ Implic. 2023 Jan 5;8:1. doi: 10.1186/s41235-022-00455-w (PMC9813289; doi:10.1186/s41235-022-00455-w)
Supplement: Supplementary file 1 — Additional file 1. A group of participants have participated in both studies, and were treated as unique observations in the ANOVA analysis for the prevalence effect. Alternative ANOVA analyses are provided here with different combinations of unique observations. [file 41235_2022_455_MOESM1_ESM.docx]

We performed ANOVA analyses with different combinations to test whether the including of unique observations qualitatively change our conclusion.

1. There were experts (*N* = 25) and novices (*N* = 33) that participated in both groups. With this specific group considered as a repeated-measure of mixed-subject design. The result is consistent with our current finding for the criteria shift. We found an interaction between the prevalence and group (*F*(1,56) = 12.88, *p* < .001, 0.062) for criteria, which was driven by statistical significance between the novices’ criteria in high and low prevalence conditions (*M* = 0.205, *t* = 2.80, *p* = .035). For d’, we found a statistical main effect for prevalence (*F*(1,56) = 15.46, *p* < .001, 0.216) and an interaction (*F*(1,56) = 6.57, *p* = .013, 0.105).


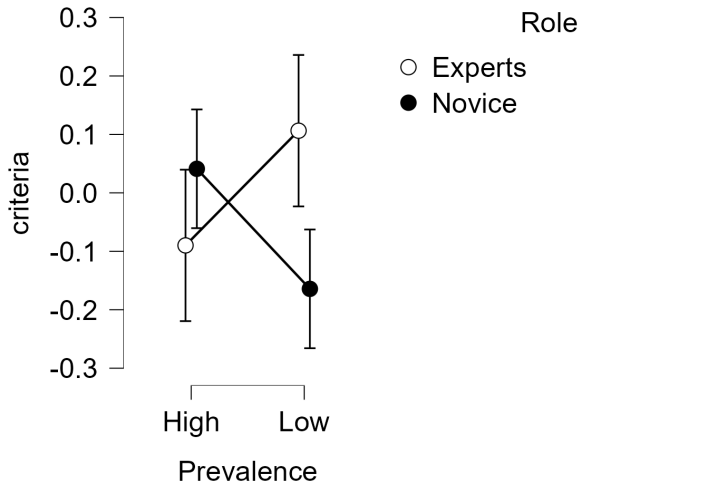

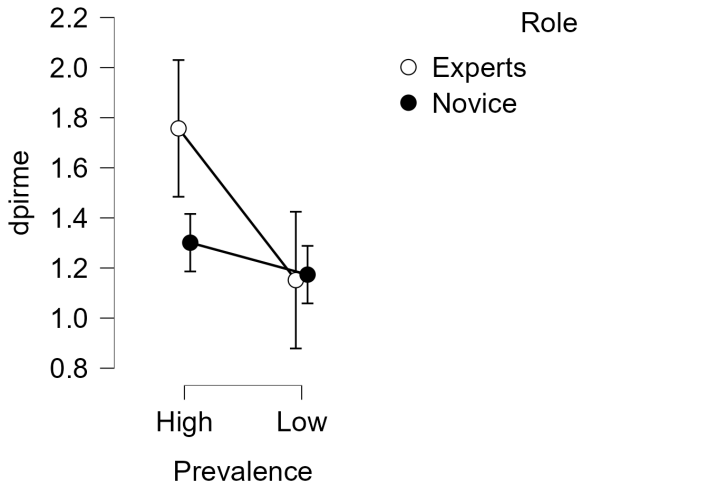


Figure 1. The average criteria and d’ with only participants who took both sections included.

1. With only unique observations included, there were 48 participants (*N*_expert_ = 11) in the high prevalence condition and 27 participants (*N_expert_* = 5) in the low prevalence condition. The ANOVA analyses reported non-significant statistical *p* value for neither the condition nor the group for both criteria and d’. We wonder if this observation can be induced by the limited number of unique experts in each observation.


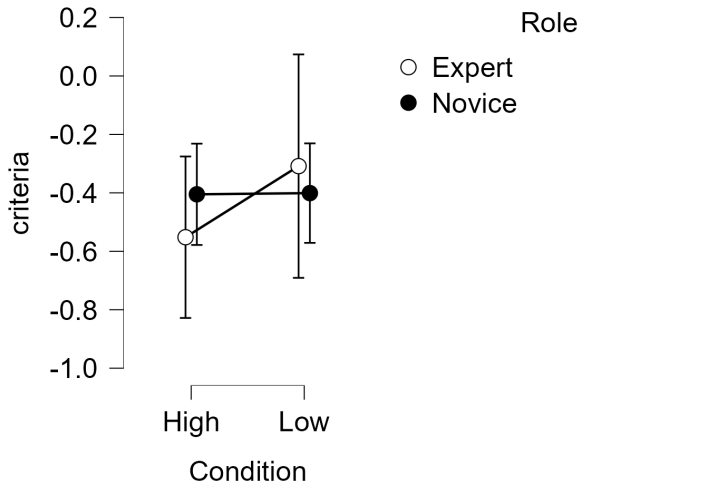


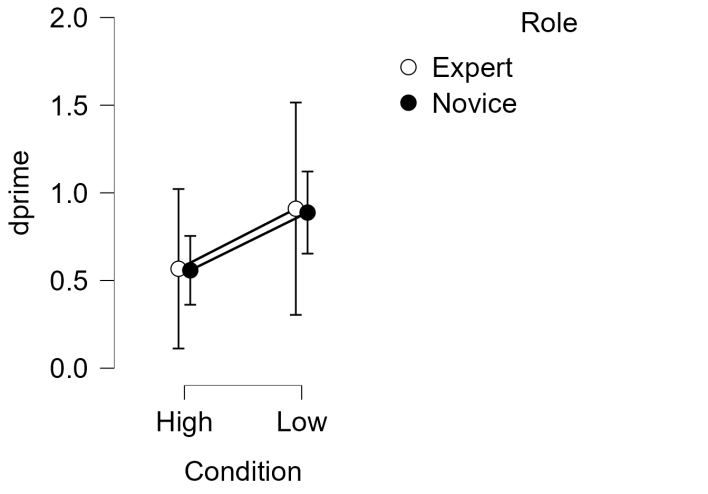


Figure 2. The average criteria and d’ with only participants who the unique section included.
